# Supplementary material for: MolOptimizer: A Molecular Optimization Toolkit for Fragment-Based Drug Design
Source: Molecules. 2024 Jan 4;29(1):276. doi: 10.3390/molecules29010276 (PMC10780997; doi:10.3390/molecules29010276)
Supplement: Supplementary file 1 [file molecules-29-00276-s001.zip › molecules-2731138-supplementary.pdf]

Supplementary Information

MolOptimizer: A Molecular Optimization Toolkit for Fragment-Based Drug Design

Adam Soffer [1,2], Samuel Joshua Viswas [1,2], Shahar Alon [3], Nofar Rozenberg [3], Amit Peled [3], Daniel Piro [3], Dan Vilenchik [4] and Barak Akabayov [1,2,*]

[1] Department of Chemistry, Ben-Gurion University of the Negev, Beer-Sheva 8410501, Israel
[2] Data Science Research Centre, Ben-Gurion University of the Negev, Beer-Sheva 8410501, Israel
[3] Department of Software Engineering, Ben-Gurion University of the Negev, Beer-Sheva 8410501, Israel
[4] School of Computer and Electrical Engineering, Ben-Gurion University of the Negev, Beer-Sheva 8410501, Israel
* Correspondence: akabayov@bgu.ac.il; Tel.: +972-8-6472-716

MolOptimizer dependencies

MolOptimizer requires several specific dependencies with precise version numbers to work smoothly and stably. This information is found in the "requirements.txt" file in our code repository's "Server" directory. These dependencies are interrelated, and installing this file ensures that any additional necessary packages are automatically handled.

For clarity and to make it easier for others to recreate our environment, we've listed the exact versions of the libraries we're using. Below are some of the key dependencies and their versions as outlined in our "requirements.txt" file:

- Django = 4.1.5
- djangorestframework = 3.14.0
- numpy = 1.24.3
- pandas = 2.0.1
- scipy = 1.10.1
- rdkit = 2023.3.2
- xgboost = 1.7.6

The architecture of MolOptimizer and the user interface (UI)

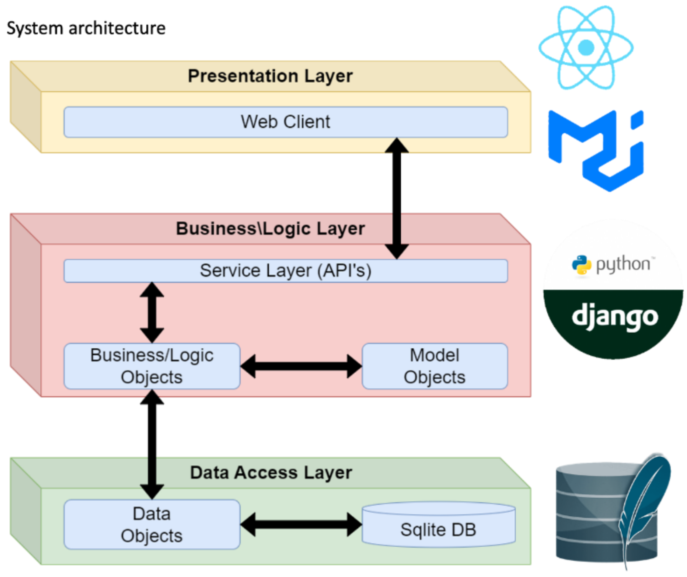

Figure S1. Architecture of MolOptimizer.

On the frontend side we used React – A JavaScript (JS) library (https://legacy.reactjs.org/) for building user interfaces and is open source component library Material UI (MUI, https://mui.com/).

We used them for 2 main reasons: 1) React JS has the advantage to be connected easily to any backend code (based on previous experience we knew to use it for our purposes). 2) We found MUI to be the best package for a great looking and a user-friendly user-interface.

On the backend side we used Django (An open source, Python-based web framework, https://www.djangoproject.com/).

The user Interface: Frontend based on React JS & Material UI. UI code is fully integrated with all backend code. The UI is adjustable, fit and tested with every available web-browser.

User management and runs management are presented in Figure S2.

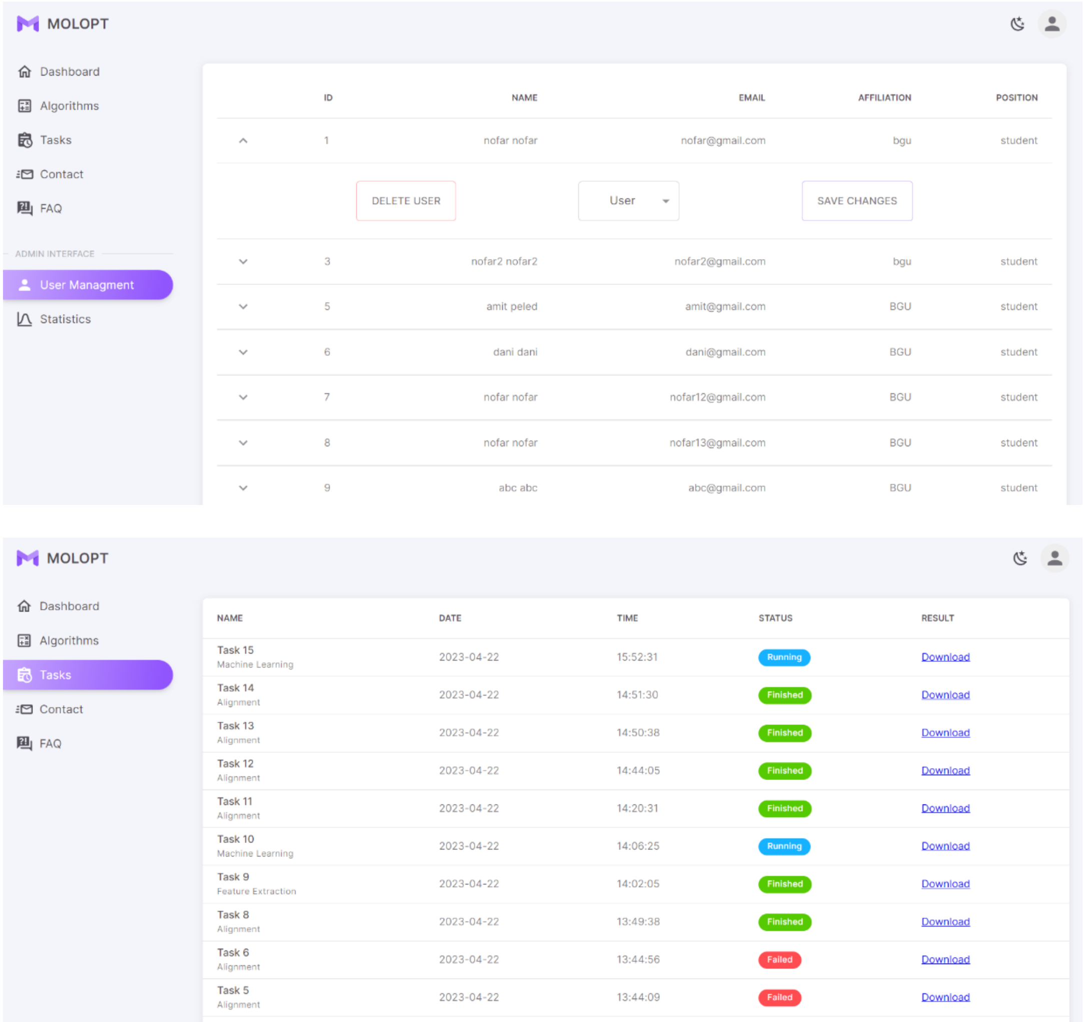

Figure S2. User and task managements.

System's Database

The database stores all the data of users and algorithm runs and is capable of saving and working with multiple users and runs in the system. The database is kept updated all the time (with new molecules that are added to the overall number of molecules).

Algorithms improvements and optimizations

Supporting the following features molecules alignment, feature extraction, and machine learning algorithms. Auto process mode is added and the system was upgraded into a thread-based multitasking application that allows parallel execution on a multiprocessing system and algorithms runs queue are designed and implemented. All these improvements were executed using Microsoft Azure® server (https://azure.microsoft.com/en-us) for better performance of MolOptimizer.

Table S1. Unique features of MolOptimizer compared to other computational tools.

FEATURE/ SOFTWARE	MOLOPTIMIZER	GRID	POCKET
**APPROACH**	Utilizes machine learning to analyze molecular features and predicts binding affinities for molecules with similar structures to the target.	Uses grids to map the energetically favorable binding sites on molecules based on physicochemical properties.	Detects pockets on the surface of proteins where ligands might bind, using geometric algorithms.
**EXAMPLE OF DATA**	Labeled small-molecule datasets with known binding values.	3D coordinates of proteins or other macromolecules, along with their associated physicochemical properties.	Protein 3D structures, typically from X-ray crystallography or NMR spectroscopy.
**REFERENCE**	*This paper*	Goodford, P.J. A computational procedure for determining energetically favorable binding sites on biologically important macromolecules. J Med Chem 28, 849-857 (1985).	Levitt, D.G., Banaszak, L.J. POCKET: a computer graphics method for identifying and displaying protein cavities and their surrounding amino acids. J Mol Graph 10, 229-234 (1992).
**WEB ADDRESS AND OTHER WEB RESOURCES**	https://molopt.online/ https://github.com/csbarak/MolOpt_Students_2023	Not available	Not available

FEATURE/ SOFTWARE	SURFNET	PASS	MMC
**APPROACH**	Analyzes the spaces between protein molecules to identify potential ligand binding sites, based on cavity detection.	Identifies putative active sites using spheres to find regions in proteins that can potentially bind to ligands.	Maps the topographical features of macromolecules to understand the surface characteristics and interaction sites.
**EXAMPLE OF DATA**	Protein 3D structures, with emphasis on the gaps and spaces within the macromolecule's surface.	Protein 3D structures and known ligand information.	Surface data from protein 3D structures, emphasizing the topology and features of the surface.
**REFERENCE**	Laskowski, R.A. SURFNET: a program for visualizing molecular surfaces, cavities, and intermolecular interactions. J Mol Graph 13, 323-330 (1995).	Brady, G.P., Stouten, P.F. Fast prediction and visualization of protein binding pockets with PASS. J Comput Aided Mol Des 14, 383-401 (2000).	Mezei, M. Mapping the surface of biomolecules. (2003). Not available as a specific paper reference; typically referenced in subsequent literature discussing molecular topography.
**WEB ADDRESS**	Not available	Not available	Not available
